# Associations between HT, BMI, and allergic rhinitis in perimenopausal women

**DOI:** 10.1186/s13223-023-00839-7

**Published:** 2023-12-19

**Authors:** Jingyi Liu, Tingting Ma, Xiaoxue Wang, Wenpei Bai, Xueyan Wang

**Affiliations:** 1 Department of Allergy, Beijing Shijitan Hospital, Capital Medical University, Beijing, 100038 China; 2 Department of Obstetrics and Gynecology, Beijing Shijitan Hospital, Capital Medical University, Beijing, 100038 China

**Keywords:** Hormone therapy, Allergic rhinitis, Perimenopausal women, Body mass index

## Abstract

**Background:**

Increasing evidence suggests that hormone therapy (HT) and obesity exert an influence on allergic rhinitis (AR). It is important to investigate the association and interactions between HT, BMI, and AR in perimenopausal women.

**Methods:**

From May 2020 to March 2021, a cross-sectional survey was completed by patients who visited the Allergy Department and Gynecology Department of Shijitan Hospital. The patients completed a questionnaire and stratified analyses by BMI in tertiles were performed. Logistic analyses were performed to evaluate the relationships between HT, BMI, and AR.

**Results:**

A total of 950 patients completed the study, among which, 393 patients were receiving HT. HT was found to be associated with increased risks for AR (OR = 1.51 [95% CI: 1.151–1.985]), asthma (OR = 3.61 [95% CI: 2.21–5.89]), and their accompanying symptoms (OR = 3.54 [95% CI: 2.146–5.831]). In lean women, the use of HT was significantly associated with a higher risks for AR (OR = 2.26 [95% CI: 1.31–3.91]), the time course of AR (OR = 2.54 [95% CI: 1.37–4.74]), hay fever (OR = 2.54 [95% CI: 1.37–4.74]), and accompanying symptoms (including canker sores, diarrhea, and stomachache) (OR = 2.26 [95% CI: 1.309–3.907]) when compared to normal or heavier weight women (course of AR: pinteraction = 0.032; hay fever; pinteraction = 0.006; accompanying symptoms: pinteraction = 0.009).

**Conclusions:**

HT can reduce the risk for AR in perimenopausal women. Lean women who used HT were at a higher risk for AR when compared to overweight women who used AR. There exists an interaction between HT and BMI that influences AR. Furthermore, HT and obesity increase the risk for AR by some common pathways, more follow-up work is needed to explore common pathways.

## Introduction

Hormone therapy (HT) has been used worldwide for a long time as a method of treating and relieving menopausal symptoms [1]. The side effects of HT on the cardiovascular system, endocrine system, female reproductive system, and the risk for breast cancer have been extensively studied. Despite growing evidence showing that HT affects respiratory health, the relationship between HT and allergic airway reactions remains unclear.

In recent years, several epidemiological studies examining the relationship between HT and allergic asthma have explored the effect of using HT on asthma development in perimenopausal women [2, 3]. The results showed that compared to patients who have never used HT, patients who have used or are currently using HT were more likely to develop asthma, when compared to patients who never used HT [4]. A multicenter cross-sectional survey conducted in northern Europe showed higher incidences of asthma, asthma symptoms, and allergy among perimenopausal women who used HT [5]. However, the role played by HT in the development of AR in perimenopausal women has been rarely reported. The association of HT with nasal allergy has only been mentioned in a few reports [5].

Allergic rhinitis (AR) is a disease of the upper airway, and shares a similar pathophysiological mechanism with asthma [6, 7]. Allergic airway diseases are considered to arise through complex interactions [8]. Obesity is a risk and aggravating factor for airway diseases, and especially for asthma and AR [9]. The inflammatory state of the airway is an important factor related to the association between obesity and airway allergy [10]. It has been reported that body weight is positively correlated with allergen sensitization threshold, which is related to the increase of proinflammatory macrophage CD11b1/CD11c1, serum IL-6 and airway eosinophils [11]. In addition, increases in the inflammatory cytokines IL-5, TNF-a and IL-10 were shown to be associated with allergic disease in obese mice [12]. Evidence shows that obesity causes immunological changes that result in a lower immunological tolerance to antigens and a disordered immune system [13]. AR, as a chronic inflammatory disease of the nasal cavity, has been confirmed to be closely related to obesity [14–16]. Body mass index (BMI) is used for obesity classification, but the association between BMI and AR remains unclear. Several studies have shown that a higher BMI is strongly associated with AR development [17, 18], whereas many other studies found no significant correlation or even a negative correlation between a high BMI and AR [13]. The association between BMI and AR is not yet well defined.

In the present study, we investigated the association between HT use and AR in perimenopausal women who visited Shijitan Hospital in Beijing, China. In addition, we also examined possible interplay between HT and BMI in association with AR.

## Methods

### Ethics statement

The study protocol was approved by the Ethics Committee of Beijing Shijitan Hospital, Capital Medical University. All subjects provided their written informed consent for study participation.

### Study population

The study was a cross sectional analysis of perimenopausal women and allergies in patients who visited the Beijing Shijitan Hospital, Beijing, China, between May 2020 and March 2021. Women aged 45–55 years were chosen to participate in this study because the mean age of onset of perimenopausal transition is 45–46 years. Briefly, the 45–55 years old women who visited the department of allergy and gynecology completed a simple questionnaire that included variables such as medical history, menopausal status, hormone use, and more. Women with artificial menopause, pregnant women, women using oral contraceptives, women older than 45 years and younger than 55 years, and women with an incomplete questionnaire were excluded from the study.

### Questionnaire

The questionnaire consisted of two parts. The first part of the questionnaire covered AR, rhinitis medications, respiratory symptoms, food reactions, drug reactions, and hay fever. Personal/family medical history and current potential treatments are included in the questionnaire. A diagnosis of allergic rhinitis required two or more of the following four symptoms: nasal congestion, nasal itching, runny nose, sneezing, and/or using a rhinitis medication, and/or having rhinitis attacks food reactions, drug reactions, during the past 1 year. The accompanying symptoms of allergic rhinitis included drowsiness, fatigue, canker sores, diarrhea, constipation, stomachache, and headache. Allergic asthma was defined as experiencing self-reported allergic asthma and/or wheeze symptoms, and/or asthma attacks, and/or having taken an asthma medication during the preceding 12 months. Seasonal onset allergic rhinitis was defined as hay fever [19].

The second part of the questionnaire covered various aspects and factors pertaining to a woman’s hormonal status. Menopause was defined as answering yes to the question “Have you had 12 consecutive months without a menstrual period (unless undergoing surgery or radiotherapy or taking medicines). The Kupperman index was used to assess the presence of perimenopausal-related symptoms, including sweating, paresthesia, insomnia, agitation, depression, suspicion, dizziness, fatigue, musculoskeletal pain, headache, palpitations, skin ants, sexual activity, and urinary tract infection. The scoring rules were as follows: asymptomatic = 0 points, mild = 1 point, moderate = 2 points, and severe = 3 points. A total score < 6 was normal, 6–15 was mild, 16–30 was moderate, and > 30 points was severe. HT was defined as answering “yes” to the question “Did you use hormone therapy after 35 years old?”. The women were also asked questions about pregnancy, their use of oral contraceptives, age of menopause, age of menarche, and date of their last menstrual bleeding.

Body mass index (BMI) was defined as self-reported weight and height and calculated as weight (kg) divided by height squared (m^2^). Questions concerning type of education and occupation were used to indicate social class. Questions about past medical history (including hypertension, hyperlipidemia, diabetes mellitus, and heart disease) were used to reflect the basic medical condition of each woman.

### Statistical analysis

A logistic regression model was used to assess associations between HT, BMI, and AR. Odds ratios (ORs) and 95% confidence intervals (CIs) were calculated in the logistic regression model. Adjustments were made for age (5-year categories), BMI (kg/m^2^), education, and occupation. Based on the World Health Organization classification, BMI was classified as lean (BMI < 20 kg/m^2^), normal weight (BMI20-25 kg/m^2^), or overweight (BMI > 25 kg/m^2^) for purposes of stratifying the HT analysis. Differences in the association between HT and AR among women with different BMIs were analyzed by incorporating the interaction term of BMI and HT into a logistic regression model. Simultaneously, the association between BMI and AR was assessed by a logistic regression model, which was stratified by HT use.

## Results

In our study, 950 eligible participants completed the questionnaire soliciting information. 41.3% (393) of the 950 women either had used or were using HT. Characteristics of the women varied depending on the use of HT. As shown in Table 1, women using HT had a lower body mass index (BMI) (*P*_BMI_ = 0.04). When compared to women who did not use HT, women who used HT had more severe menopausal symptoms (Kupperman index = 30 points) (*P*_kupperman index_ = 0.0001) and were more likely to seek medical attention as a result. Women who used HT were less likely to have hypertension, but had higher incidences of heart disease, more diabetes mellitus, and more hyperlipidemia (*P*_heart disease_ < 0. 0001, *P*_diabetes mellitus_ < 0 0.0001, *P*_hyperlipidemia_ < 0 0.0001).

**Table 1:** Demographic and baseline characteristics of study populations

	HT (n = 393)	No HT (n = 557)	*P* value
Age (year)	49.86 ± 2.67	49.96 ± 2.61	0.580
BMI (kg/m^2^)	21.76 ± 2.67	22.14 ± 2.96	0.041
Education			0.817
Less than elementary	4 (1.02%)	8 (1.44%)	
Less than high school	21 (5.34%)	36 (6.46%)	
Less than college	97 (24.68%)	131 (23.52%)	
More than college	271 (68.96%)	382 (68.58%)	
Occupation			0.958
Office work	134 (34.1%)	187 (33.57%)	
Manual work	238 (60.56%)	338 (60.68%)	
Housewife	21 (5.34%)	32 (5.75%)	
Kupermann index(%)			< 0.0001
0 ~ 6 points	28 (7.12%)	82 (14.72%)	
7 ~ 15 points	79 (20.1%)	165 (29.62%)	
16 ~ 30 points	191 (48.6%)	251 (45.06%)	
30 points	95 (24.17%)	59 (10.59%)	
Hypertension (%)	243 (61.83%)	270 (48.47%)	< 0.0001
Heart disease (%)	76 (19.34%)	64 (11.49%)	< 0.0001
Diabetes mellitus (%)	119 (30.28%)	100 (17.95%)	< 0.0001
Hyperlipemia (%)	114 (29.01%)	92 (16.52%)	< 0.0001

A study of the relationship between AR and endogenous estrogen-related factors showed that age at menarche was not associated with AR. A stratification by number of pregnancies showed that the number pregnancies or lack of a pregnancy was also not related to AR (Table 2). In postmenopausal women, AR was not associated with age at menopause or postmenopausal duration.

**Table 2:** Odds ratios for the association between each endogenous-related hormone factors and prevalence of allergic rhinitis in perimenopausal women

	Allergic rhinitis (+) (n = 316)	Allergic rhinitis (−) (n = 634)	OR (95% CI)	*P* value
Age at menarche, y				
<14	130 (33.59%)	257 (66.41%)	1.00	
>=14	186 (33.04%)	377 (66.96%)	0.99 (0.75, 1.32)	0.957
Age at menopause, y				
>50	45 (33.83)	88 (66.17)	1.00	
<=50	48 (24.87)	145 (75.13)	0.65 (0.39, 1.08)	0.099
Postmenopausal duration, m				
<6	166 (35.93)	296 (64.07)	1.00	
>=6	93 (35.63)	168 (64.37)	0.91 (0.65, 1.28)	0.603
Number of pregnancy				
0	32 (32.65)	66 (67.35)	1.00	
1 ~ 2	190 (33.39)	379 (66.61)	0.84 (0.52, 1.35)	0.472
>2	94 (33.22)	189 (66.78)	0.76 (0.45, 1.29)	0.312

As shown in Table 3, an analysis of the association between HT and AR showed that women using HT more often reported having AR (OR = 1.511 [95% CI: 1.151–1.985]). HT was notably associated with AR symptoms, including itching, blockage, and rhinorrhea. The symptom of sneezing was not significantly increased in HT women. In addition, we calculated the number of women with three or more rhinitis symptoms and found that women with three or four rhinitis symptoms were significantly more likely to be using HT (OR_three symptoms_, 1.504; 95% CI: 0.932–2.426; OR_four symptoms_, 1.795; 95% CI: 1.307–2.465). Other symptoms such as hyposmia and sore throat were also clearly increased in women receiving HT. However, there was no significant difference between the courses of AR and hay fever in the two groups. We also assessed the association between allergic asthma and other accompanying symptoms and HT. The results showed that the incidence of allergic asthma was significantly increased in HT women. Moreover, accompanying symptoms (including canker sores, diarrhea, and stomachache), food reactions, and drug reactions were also more common in women using HT (OR_accompanying symptoms_ = 3.54; 95% CI: 2.15–5.83; OR_food allergies_ = 3.82; 95% CI: 2.85–5.13; OR_drug allergies_ = 4.16; 95% CI: 3.02–5.73) (Table 4).

**Table 3:** Allergic rhinitis and hay fever according to use of HT among 950 women

	HT (n = 393) (%)	No HT (n = 557) (%)	OR (95% CI)	*P* value
Allergic rhinitis**#**	51.83%	43.82%	1.511 (1.15,1.99)	0.003 (8.849)
Itching	36.39%	25.85%	1.641 (1.24,2.17)	0.000 (12.127)
Blockage	31.81%	25.13%	1.389 (1.04,1.85)	0.024 (5.100)
Sneezing	31.30%	26.57%	1.259 (0.95,1.67)	0.112 (2.525)
Rhinorrhea	32.3%	23.9%	1.522 (1.14,2.03)	0.004 (8.253)
Two or more nasal symptoms*	5.85%	7.36%	0.782 (0.46,1.33)	0.361 (0.834)
Three nasal symptoms	9.4%	6.5%	1.504 (0.93,2.43)	0.093 (2.830)
Four nasal symptoms	26.2%	16.5%	1.795 (1.31,2.47)	0.000 (13.267)
Hyposmia	23.66%	15.62%	1.675 (1.21,2.32)	0.002 (9.710)
Sore throat	24.68%	15.98%	1.723 (1.25,2.38)	0.001 (11.085)
Course of disease	77.86%	81.15%	0.817 (0.59,1.12)	0.214 (1.514)
Hay fever**##**	35%	35.3%	0.989 (0.67,1.46)	0.957 (0.003)

#The diagnosis of allergic rhinitis requires nasal congestion, nasal itching, runny nose, sneezing symptoms, and take allergic rhinitis medication and/or rhinitis attacks last 12 months

*Two or more nasal symptoms included: Itching, Sneezing, Blockage, Rhinorrhea

##Hay fever was defined as the seasonal onset of allergic rhinitis

**Table 4:** Allergic asthma and accompanying symptom according to use of HT among 950 women

	HT (n = 393)(%)	No HT (n = 557)(%)	OR (95% CI)	*P* value
Allergic asthma**	14.50%	4.70%	3.610 (2.21,5.89)	0.000 (29.308)
Accompanying symptoms***	13.74%	4.31%	3.538 (2.15,5.83)	0.000 (27.198)
Drowsiness	51.4%	55.3%	0.855 (0.66,1.11)	0.236 (1.407)
Fatigue	61.1%	60.1%	1.040 (0.80,1.35)	0.774 (0.083)
Canker sores	39.7%	32.7%	1.356 (1.04,1.77)	0.026 (4.954)
Diarrhea	33.2%	20.3%	1.949 (1.45,2.62)	0.000 (19.777)
Constipation	38.2%	33.9%	1.202 (0.92,1.57)	0.180 (1.802)
Stomachache	23.7%	13.3%	2.023 (1.44,2.84)	0.000 (17.131)
Headache	17.6%	19.9%	0.856 (0.61,1.19)	0.358 (0.843)
Food allergy recation	45.55%	17.95%	3.823 (2.85,5.13)	0.000 (84.580)
Drug allergy	38.17%	12.92%	4.158 (3.018,5.729)	0.000 (81.982)

**Allergic asthma: Allergic asthma was defined as self-reported allergic asthma and/or wheeze symptoms and/or asthma medication and/or asthma attacks in the last 12 months

***Accompanying symptoms included: Drowsiness, Fatigue, Canker sores, Diarrhea, Constipation, Stomachache, Headache

After stratifying for BMI (Table 5), we analyzed the effects of BMI and HT on AR. Our data showed that HT was not significantly associated with rhinitis or rhinitis symptoms in normal weight women and overweight women. However, in lean weight women, the use of HT was strongly associated with the risk for AR, the course of AR, hay fever, and accompanying symptoms. AS shown in Table 5, in perimenopausal women with BMI < 20 kg/m^2^, the incidence of AR increased significantly after HT use compared with no HT use (P 0.003).The total number of women receiving HT and who had rhinitis lasting for 3 years or less was significantly less than the number of lean women who did not use HT. The associations between HT and course of AR, course of disease, hay fever, and accompanying symptoms in the lower BMI tertile were clearly more obvious than the relevant associations in the medium and upper tertiles (p_interaction_ = 0.032, 0.006, and 0.009, respectively). The interactions between BMI and HT were significant with regard to their associations with AR.

**Table 5:** Allergic rhinitis in 590 women according to use of MHT, stratified by BMI

	HT (n = 393)(%)	No HT (n = 557)(%)	OR (95% CI)	*P* value
BMI <20 kg/m^2^ (n = 235)	26.46%	23.52%	1.170 (0.87,1.58)	0.300 (1.073)
Allergic rhinitis**#**	45.19%	26.72%	2.262 (1.31,3.91)	0.003 (8.710)
Three or more nasal symptoms*	5.78%	6.87%	0.944 (0.33,267)	0.914 (0.012)
Course of disease	68.27%	86.26%	0.343 (0.18,0.65)	0.001 (11.042)
Hay fever	32.69%	16.03%	2.544 (1.37,4.74)	0.003 (8.978)
Accompanying symptoms**	45.19%	26.72%	2.262 (1.31,3.91)	0.003 (8.710)
20 kg/m^2^ < BMI < 25 kg/m^2^ (n = 596)	62.34%	63.02%	0.972 (0.74,1.27)	0.832 (0.045)
Allergic rhinitis**#**	34.69%	28.77%	1.315 (0.93,1.87)	0.125 (2.355)
Three or more nasal symptoms*	5.71%	5.98%	0.952 (0.47,1.91)	0.891 (0.019)
Course of disease	80.82%	80.06%	1.049 (0.70,1.58)	0.818 (0.053)
Hay fever	22.04%	18.80%	1.221 (0.82,1.83)	0.332 (0.940)
Accompanying symptoms**	34.69%	28.77%	1.315 (0.93,1.87)	0.125 (2.355)
BMI > 25 kg/m^2^ (n = 119)	11.20%	13.46%	0.810 (0.55,1.21)	0.298 (1.083)
Allergic rhinitis**#**	45.45%	37.33%	1.399 (0.66,2.98)	0.383 (0.760)
Three or more nasal symptoms*	6.82%	14.67%	0.426 (0.11,1.62)	0.200 (1.646)
Course of disease	84.09%	77.33%	1.549 (0.59,4.10)	0.375 (0.787)
Hay fever	20.45%	25.33%	0.758 (0.31,1.86)	0.545 (0.367)
Accompanying symptoms**	45.45%	37.33%	1.399 (0.66,2.98)	0.383 (0.760)

Interaction in effect of HT on AR between lean and normal/overweight women pinteraction = 0.000

Interaction in effect of HT on Course of disease between lean and normal/overweight women pinteraction = 0.001

Interaction in effect of HT on hay fever between lean and normal/overweight women pinteraction = 0.000

Interaction in effect of HT on Accompanying symptoms between lean and normal/overweight women pinteraction = 0.000

## Discussion

The influence of HT and obesity on AR has been extensively discussed [20]. A better understanding of how HT and BMI impact AR could aid in managing rhinitis symptoms in perimenopausal patients and thereby considerably reduce the social and economic burden of the disease. The perimenopause is an important period in the life course of women. Instability of the hypothalamic-pituitary ovarian axis (HPO) in perimenopausal women leads to fluctuations in sex hormone levels, various discomfort symptoms, as well as endocrine disorders, disorders of glucose and lipid metabolism, hypertension, and heart disease [21, 22]. All of these disorders prompt perimenopausal women to seek medical attention and professional hormone therapy to relieve their symptoms.

The present study enrolled 950 women aged 45–55 years, of which 393 (41.3%) were using HT at the time of the study. HT is a potent treatment for menopausal symptoms and has been previously used to reduce coronary heart disease (CHD) and mortality [23]. However, HT can cause side effects such as breast tenderness, mood changes, and uterine bleeding. Of particular concern, HT increases the risks for cancer and venous thromboembolism [24, 25]. Recent studies have found that HT increases the risk for asthma and asthma symptoms in women. However, rarely studies have reported the effects of HR on perimenopausal women, and especially its effects on body mass index. In our study, women who used HT had a lower body mass index, more menopausal symptoms, a lower rate of hypertension, higher rates of heart disease, type 2 diabetes, and hyperlipidemia, but a lower prevalence of hypertension. These results are similar to those in previous studies [26]. However, the effects of hormone therapy on blood pressure are controversial [27, 28]. In our study, there was a significant reduction in the incidence of hypertension among perimenopausal women receiving HT, which we considered to be related to hormone therapy improving the biological effects of nitric oxide (NO) and reducing the effects of angiotensin II, thereby causing vasodilation and leading to a decrease in blood pressure [29].

Several studies have shown that sex hormones are associated with rhinitis [30]. Female sex hormone receptors expressed by nasal epithelial cells are correlated with the severity of rhinitis symptoms [31]. The use of oral hormones was found to induce inter-epithelial edema and histiocytic proliferation in human nasal mucosa [32]. Furthermore, circumstantial evidence for the effect of female sex hormones on rhinitis suggests that the menstrual cycle and pregnancy can cause rhinitis symptoms [33]. In this study, the prevalence of AR was significantly higher among HT users. However, the relationship between the two was independent of age at menarche, number of pregnancies, and age at menopause. HT not only causes AR and asthma, but also impairs the immune system and leads to other allergic symptoms. In our research, we found that perimenopausal women who used HT had more asthma, more accompanying symptoms, and more often developed food and drug reactions. When taken together, these results indicated that the duration of endogenous estrogen exposure was not associated with nasal mucosal sensitization status in premenopausal women treated with HT, but might be associated with systemic allergen sensitization status [34, 35].

Obesity exacerbates HT-induced inflammation, activates inflammatory cells, and thereby contributes to the severity of AR [18, 36, 37]. This study showed that the proinflammatory effect of IR in high BMI may be the cause of AR, that is, the direct effect of HT on AR appears to be offset by the improvement in IR-dependent AR in obese women. The risks for AR and relevant symptoms related to HT use were significantly greater in lean women than in normal weight and heavier women. The possible mechanism for this finding is that in lean women, HT exerts a direct pro-inflammatory effect on the organism and thus contributes to the development of AR, compared with lower levels of insulin resistance. However, in heavier women, this effect may be counteracted by a reduction in HT-related IR. Obesity leads to insulin resistance (IR) and inflammation [38, 39], and previous studies suggest that the association between AR and BMI may be due to the proinflammatory effects of IR [40, 41]. The same mechanism has been described for breast cancer, where the risk for breast cancer among HT users was greater in lean women [42, 43], suggesting a complex interplay between sex hormones, fatty tissue, and metabolism.

This is the first clinical study to examine the connection between HT and AR and investigate the relationship between obesity and AR in perimenopausal women. At the same time, this is the first study to look at the interaction between HT and obesity in AR. However, our study does have some limitations. First, we examined age, education, and occupation as potential confounders, but did not examine numerous other variables such as smoking history and living environment (rural or urban). Further studies with large samples sizes should be conducted to investigate the effect of additional related variables in the future. Second, the specific mechanisms of interactions among HT, AR, and BMI were not thoroughly investigated. Additional studies with large sample sizes will be needed to explore the mechanism of interaction between HT and AR. Third, we did not know what type of HT was used or when it was used by the patients. Therefore, we were unable to know the order of AR and HT, and the effect of specific drugs on the disease. However, in some previous studies, investigators did not observe a difference between the effects of various hormones on lower airway anaphylaxis [44]. Although the causes and underlying mechanisms of HT and AR require further exploration, this is the first study to evaluate the relationships between HT, BMI, and AR prevalence in perimenopausal women.

## Conclusion

In this present study, we found an association between HT and AR, and confirmed the association between BMI and AR. HT was associated with an increased risk for AR in lean women when compared with normal weight and overweight women. Our results suggest that IR, inflammation, obesity, and HT may be involved in the pathogenesis of AR and allergy by acting through partly common pathways. Allergic diseases may be a side effect of HT. Therefore, future studies of allergic airway disease and BMI should take a person’s current hormonal status into account.

## Data Availability

The datasets used and/or analysed during the current study are available from the corresponding author on reasonable request.

## Abbreviations

AR: Allergic rhinitis
HT: Hormone therapy
BMI: Body mass index
HPO: Hypothalamic-pituitary ovarian axis
CHD: Coronary heart disease
NO: Nitric oxide
IR: Insulin resistance
